# Histological ageing of fractures in infants: a practical algorithm for assessing infants suspected of accidental or non‐accidental injury

**DOI:** 10.1111/his.13850

**Published:** 2019-04-29

**Authors:** Anie Naqvi, Emma Raynor, Anthony J Freemont

**Affiliations:** ^1^ University of Manchester Medical School Manchester UK; ^2^ School of Biological Sciences University of Manchester Manchester UK

**Keywords:** ageing, fracture, histology, infant

## Abstract

**Aims:**

This study is the first to systematically document histological features of fractures of known age in infants (≦12 months). It has been used to develop a tabulated database specifically to guide histopathologists to age fractures in children considered to have suffered accidental or non‐accidental injury (NAI). Currently in the United Kingdom there are insufficient pathologists with experience in histological ageing of fractures to meet the medicolegal need for this examination. This study provides a practical tool that will allow those skilled paediatric and forensic pathologists currently involved in assessing infants for evidence of accidental or non‐accidental injury a basis for extending their assessment into this area of unmet need.

**Methods and results:**

One hundred and sixty‐nine fractures of known age at death were obtained from 52 anonymised infants over a period of 32 years (1985–2016 inclusive). Sections stained using haematoxylin and eosin (H&E) and Martius scarlet blue (MSB) were used to identify specific histological features and to relate them to fracture age. In 1999 the data were entered into a tabulated database for fractures accumulated between from 1985 to 1998 inclusive. Thereafter cases were added, and at 2‐yearly intervals the accumulated data were audited against the previous database and adjustments made.

**Conclusions:**

This paper describes the final data set from the 2017 audit. The study was terminated at the end of 2016, as there had been no material changes in the data set for three consecutive audits.

## Introduction

Recognition of fractures in life and at autopsy is a key element in the assessment of infants believed to have suffered accidental or non‐accidental injury.^1^

Radiological skeletal survey (SS) is the current method of choice in detecting fractures in life.^2^ Follow‐up SS increases the likelihood of fracture detection in live children,^2^, ^3^ but as progressive healing does not occur post‐mortem, radiological detection of recent fractures post‐mortem is more challenging,^4^ although more sensitive techniques that require doses of radiation that could not be given to live infants [e.g. whole‐body computerised tomography (CT)] can be employed.^2^, ^5^ However, while radiology detects overt and unsuspected fractures in dead infants, many may be missed.^6^, ^7^, ^8^, ^9^ Significantly, it is fractures that most closely correlate with NAI (e.g. posterior rib and metaphyseal) that are commonly missed.^10^, ^11^, ^12^ Further evidence of fracture can be obtained at autopsy,^9^, ^13^ but naked eye examination also misses key fractures.

Part of the full investigation of fractures detected/suspected at autopsy is histological examination of the bone in order to:

- decide if bone injury has occurred;
- age fractures relative to the time of death; and
- determine whether there is an abnormality of the bone (e.g. osteogenesis imperfecta, osteoporosis, vitamin D deficiency) that might make bones more easily fractured.

It is accepted that post‐mortem histological examination of infant bones can detect fractures not seen radiologically.^9^, ^11^, ^13^, ^14^ Optimising detection of fractures is key, as multiple fractures and fractures of certain bones/parts of bones (e.g. posterior ribs, limb bone metaphyses) carry a high correlation with NAI and have causational implications.^10^, ^12^, ^14^, ^15^, ^16^ The causational significance of demonstrating fracture types is well reported,^11^, ^14^, ^17^ and we recently demonstrated^13^ how routine sampling of apparently unfractured bones might disclose key, unsuspected fractures.

Ageing fractures is complementary to their detection. In the absence of underlying bone disease, fractures of multiple ages strongly correlate with NAI. Distinguishing ante‐ from post‐mortem fractures and placing fractures within a temporal landscape can assist in testing witness statements and contribute to understanding the chronology of events prior to death. However, while there are time‐lines for the processes of fracture healing in experimental animals and experiential evidence of similar timelines in humans,^9^, ^18^, ^19^, ^20^ there are no systematic studies documented in such a way as to help histopathologists with limited knowledge of histological bone biology to age fractures in infants.

Here we describe the results of a 32‐year study to generate a practical tabular database allowing recognition of histological characteristics of fracture healing for assessing fracture age.

The starting point was a data set of eligible fractures from cases received in the University of Manchester's Osteoarticular Pathology laboratory (UMOAP) from January 1985 to December 1998, analysed in 1999. Thereafter cases were added and the accumulated data analysed every 2 years. This paper describes the final data set from the 2017 audit. The study was terminated at the end of 2016, as there had been no material changes in the data set for three consecutive audits (i.e. since 2011).

## Materials and Methods

Fractures from infants (≦12 months) were sent to UMOAP by forensic and paediatric pathologists. In every case employed in this study the age of the fracture (the interval between fracturing event and death) was documented. This took the form of a precise history and fractures confirmed radiologically. It is very uncommon to acquire such evidence in cases of NAI, but less so in real or claimed accidental injury. All cases were documented anonymised of all patient details except age and fracture site (bone and position within that bone) by one specialist osteoarticular pathologist (A.F.). They included single and multiple fractures from the same individual, including individuals with fractures of different known ages.

The data set analysed in 1999 consisted of 99 fractures from 27 infants. During the subsequent 18 years a further 70 fractures were added from 25 infants. The rarity of having timed fracturing events in cases of NAI, and accidental injuries being uncommon in this age group, is reflected in there being only 169 fractures in this study from post‐mortem examination of ~3200 fractures of infants in UMOAP during the same period. In some cases, in any one infant, evidenced timing was available for a proportion of the fractures only and in some for more than one episode of fracturing.

All tissue was decalcified in 10% formic acid under radiological control, and resultant tissue blocks were sectioned and stained with H&E and MSB.

Underlying bone disease that might affect the rate of healing (e.g. osteoporosis/vitamin D deficiency) was excluded histologically.

From 1985, all eligible fractures were documented for the presence of histological features^19^, ^21^, ^22^, ^23^ that, from animal studies and reported cases in adults and infants, reflected the progressive processes of fracture healing. These were:

- Haemorrhage in and around the fracture line
- Visible strands of fibrin in/adjacent to the fracture line
- Polymorph infiltrate between the bone ends and within haemorrhage
- Macrophage infiltrate in the fracture line/adjacent marrow
- Granulation tissue formation
- Osteocyte loss
- Osteoclasts removing cortical/trabecular bone at fracture sites
- Condensation of mesenchyme
- Early woven bone formation
- Distinct bone trabeculae and cartilage nodules
- Calcification
- Fracture union
- Organisation of primary callus and deposition of lamellar bone
- Normal bone structure restored

Data were tabulated to show the time prior to death at which each feature was seen and the proportion of fractures from each time‐point showing the feature. The time‐points were largely self‐selecting by the number of fractures, each time‐point having to have at least four fractures from at least two infants.

After 1998, all data (including new cases added in period) were summated every 2 years and an audit performed comparing the ‘new database’ with that of 2 years earlier. While this led to changes in consecutive databases, most of the changes were caused by the increasing number of samples resulting in increased numbers of time‐points. However, from the 2011 audit (cases to December 2010), no changes at all were made to the database despite adding further cases. As per the original protocol, the study was terminated after three successive unchanged 2‐yearly audits at the end of 2016.

## Results

The age of the infants did not conform to a conventional Gaussian distribution, two‐thirds being less than 6 months old. The median was 4.1 months and the range 3 weeks to 11.9 months (M:F = 28:24).

### Data from the Tabulated Analyses

**Figure 1:**
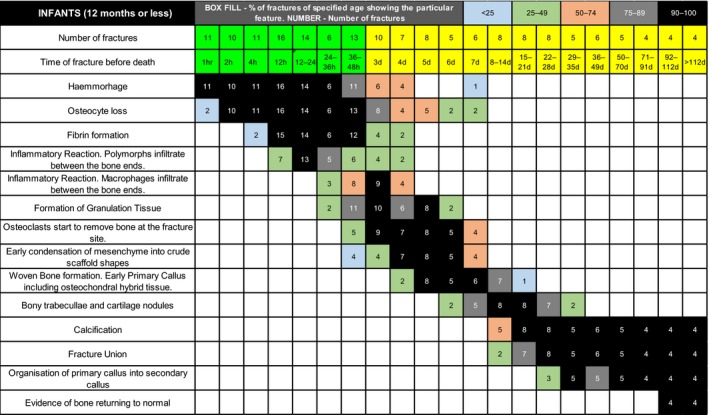
Graphic table showing the presence of histological features in fractures of different age. The proportion of fractures of a specific age showing the histological feature is represented by different colours of the boxes (see key in top row of figure). Number of cases showing the feature is also given. Timings measured in: hours = green‐headed columns (2–8); and days = yellow‐headed columns (9–22).

The full 2017 data set recording the appearance, presence and disappearance of each histological feature and the number of cases showing that feature tabulated against time from death in Figure 1.

The data indicate a reproducible and universal sequence of histologically defined events as healing progresses, including haemorrhage; clot formation; inflammatory cell infiltration; granulation tissue formation; stimulation of mesenchymal stem cells; production of matrix: appositional (on pre‐existing bone surfaces), *de‐novo* (in fibrous tissue) or endochondral (on surface of cartilage‐like callus); osteoclastic/osteoblastic bone remodelling with lamellar bone formation; and return of a more normal structure. Features of progression varied between cases, presumably through biological variation, but were remarkably consistent in different fractures from the same individual.

### A Review of the Histological Features Originally Used for the Study

#### Haemorrhage

**Figure 2:**
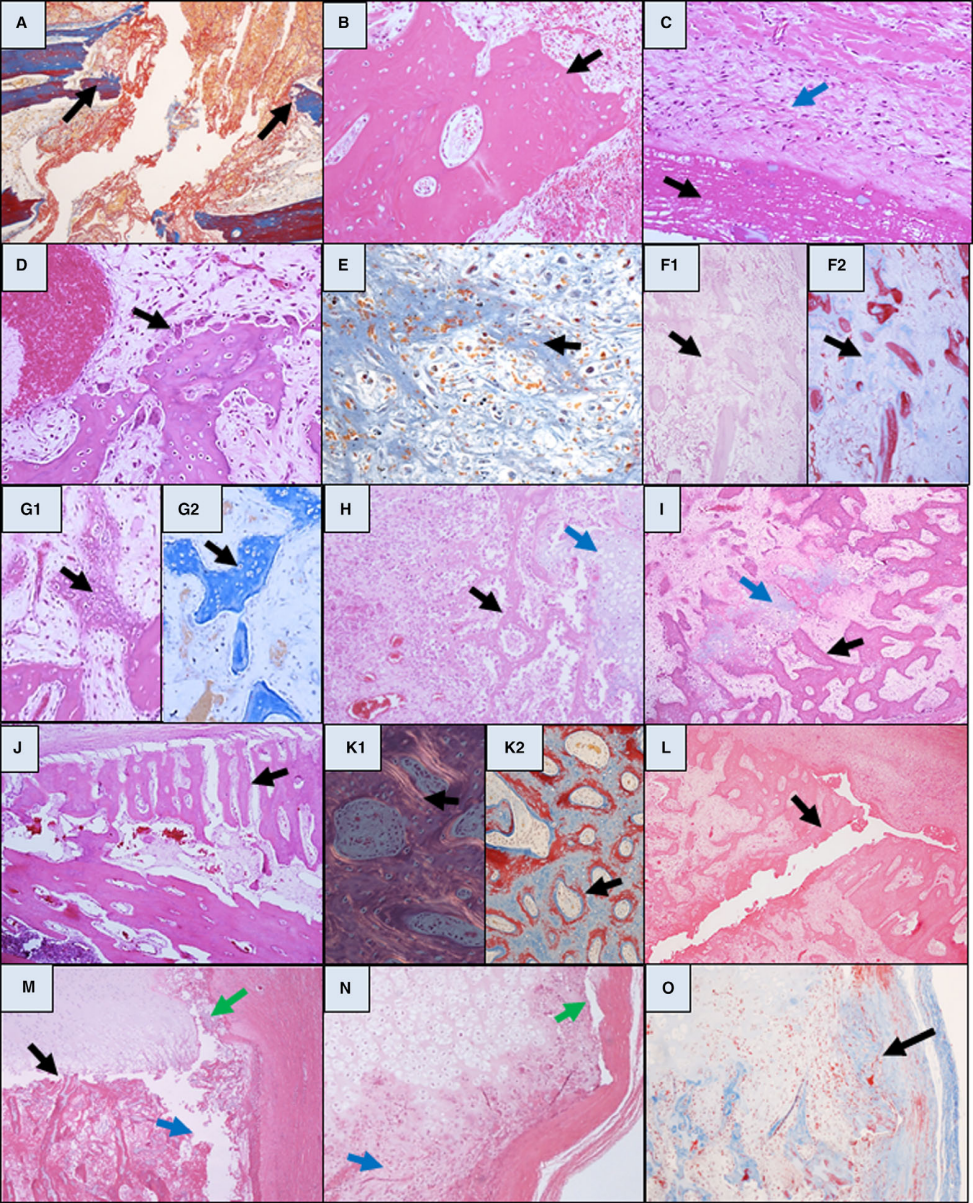
Histological sections [haematoxylin and eosin/Martius scarlet blue (H&E/MSB)] from infant fractures of specified ages. **A**, MSB (24 h). Haemorrhage [yellow, red blood cells (RBC)] and fibrin (red fibres) in fracture line (arrowed). **B**, H&E (2 h). Osteocyte lacunae mostly empty. **C**, H&E (3 days). Surface fibrin (black arrow) and periosteal cell proliferation (blue arrow). **D**, H&E (3 days). Osteoclasis of native metaphyseal periosteal bone. **E**, MSB (4 days). Granulation tissue/mesenchymal condensation (arrowed). **F1**, H&E and F2) MSB (4 days). MSB makes early osteoid (arrowed) more visible and distinguishable from fractured native bone (red on MSB); valuable when scanning multiple bones for fracture. **G1**, H&E (5 days) and **G2**, MSB (8 days). New hybrid matrix forming crude trabeculae (arrowed). **H** (9 days), **I** (22 days), H&E. Trabeculae of woven osteoid (black arrows) and cartilage nodules (blue arrows). **J**, H&E (24 days) periosteal callus (trabeculae at right angles to surface of native bone) and medullary callus (**I2**) showing variation in colour across single trabeculae. **K1**, H&E polarised light and **K2**, MSB (40 days) surface lamellar bone. Distinct lamellae in polarised light and irregular red colour on MSB (arrowed). **L**, H&E. 21‐day fracture with 12‐h refracture extending completely through pre‐existing bridging periosteal callus (arrowed). **M**, H&E (12 h). Metaphyseal fracture. T‐shaped fracture line dividing metaphyseal cartilage from primary spongiosa (black arrow), extending through adjacent medullary bone (blue arrow), stripping perichondrium (green arrow). **N**, H&E (4 days). Metaphyseal fracture with granulation tissue in medulla (blue arrow) and subperichondrial space (green arrow). **O**, MSB (5 days). Fracture at the growth plate involving cortex with a healing response consisting of medullary granulation tissue and early osteoid formation (arrowed) in the cortical fracture line.

The presence of RBC within the fracture line (Figure 2A) is used to assess if a fracture is ante‐ or post‐mortem [it is generally accepted that post‐mortem fractures (including CPR‐induced) do not bleed significantly]. Notably, some fractures occurring shortly before death had no RBC in the cortical component of the fracture line, but showed haemorrhage into, and distorting, immediately adjacent marrow and/or periosteal/subperiosteal tissues. Never having seen haemorrhage displace marrow or penetrate the periosteum in post‐mortem or CPR‐induced fractures, or loss of RBC in the presence of identifiable fibrin, we hypothesise that RBC in unclotted blood may be lost from the cortical component of the fracture site as part of tissue removal and processing, but that this does not indicate a post‐mortem fracture. Based on these findings, we have redefined ante‐mortem haemorrhage to include significant medullary or periosteal haemorrhage with or without RBC in the cortical fracture line.

#### Osteocyte loss

In keeping with the literature,^18^ osteocyte loss occurred in fractures at 7 days. However, our findings highlight osteocyte necrosis in cortical/trabecular bone at fracture edges from 1 h following fracture (see Figures 1 and 2B). Our experience is that this pattern of osteocyte loss is never seen in post‐mortem fractures, thus osteocyte loss indicates an ante‐mortem fracture.

#### Fibrin formation

Distinct strands (pink on H&E, orange‐red on MSB) are universally seen at 12 h, increasing appreciably over 24 h (Figure 2A,C), and thereafter disappearing as other processes become dominant.

#### Polymorph infiltrate

This could be difficult to appreciate in its early stages because of marrow and blood neutrophils, but was very noticeable at the periphery of areas of haemorrhage by 18 h. It is short‐lived.

#### Macrophage infiltrate

Macrophages appeared between bone ends as blood clot transitioned into true callus. It is not a universal finding, and adds little to the assessment of ageing in fractures.

#### Granulation tissue formation

Fibroblast ingrowth was the earliest evidence of granulation tissue formation, followed by capillary proliferation and increasing collagen deposition.

#### Osteoclasis

Osteoclasis is variable. There was a consistent burst of osteoclastic bone erosion throughout the fracture from days 3–7. However, in infants with metaphyseal fractures, cortical osteoclasis occurred much earlier (24–36 h; Figure 2D).

#### Mesenchymal condensation and woven bone formation

Mesenchymal condensation and stromal cell proliferation occurred in the medulla (Figure 2E,F) and periosteum (Figure 2C). This was followed by more distinct non‐mineralised bone‐like tissue formed into crude trabeculae, on bone surfaces and *de‐novo* (Figure 2G). Some had a hybrid osteochondral morphology, consistent with previous findings.^24^ While visible on H&E‐stained sections, new osteoid was more obvious with MSB, standing out from background tissue as pale blue cords (Figure 2F,G). It was seen universally by 5 days, increasing to 14 days.

#### Bony trabeculae and cartilage nodules

Transition from indistinct cords of woven osteoid/osteochondral material to trabeculae of bone and cartilage nodules (Figure 2H,I) occurred by 8–14 days, increasing to 21 days. Cartilage was particularly prominent in rib fractures and occasionally was the only periosteal callus material adjacent to fracture lines.

#### Calcification

Calcification leads to variation in the colour within osteoid trabeculae in H&E sections (pink, mottled ‘dirty’ grey/pink, or blue/pink) (Figure 2I,J). It was seen in most fractures between 15 and 21 days and in all by 22–28 days.

#### Fracture union

All cases showed bridging periosteal bony/cartilaginous fracture callus by 22–28 days.

#### Early remodelling

Deposition of lamellar bone on pre‐existing woven bone or cartilage nodules was taken as the first sign of remodelling towards a normal bone microstructure. Best identified in polarising light (Figure 2K1), lamellar bone was never seen before 28 days and always by 36 days, increasing thereafter. Lamellar bone also exhibited surface osteoblasts and appeared red/blue on MSB (Figure 2K2).

#### Evidence of bone restoration towards a normal structure

Return towards the structure of ‘normal’ bone was clearly established by 92 days after fracture, after which differential histological ageing became difficult.

#### Mobile fracture sites

Mobile fractures, particularly rib fractures, often showed healing reactions of different ages. We hypothesise that this is due to recurrent trauma at the fracture site consequent upon motion. In this setting, we found that the periosteal healing reaction away from the fracture line most accurately reflected the fracture's age.

#### Refractures

Refractures are recognised by a fracture line passing completely through existing fracture callus (Figure 2L). While the complexity of combined fracture responses means that it is not always possible to age a refracture, by definition the presence of refracture indicates a second, later fracturing event.

#### Metaphyseal fractures

Many fractures were metaphyseal, typically of anterior and posterior ribs and the long limb bones. The metaphysis contains a cartilage/bone interface, a site of weakness. Forceful pulling and twisting can cause fractures along the interface, which are well described in the literature.^11^, ^14^, ^17^, ^25^ There are two features that need stressing when ageing metaphyseal fractures:

- fractures through the growth plate cartilage do not show features of repair and cannot be aged; and
- most metaphyseal fractures propagated through bone at their periphery. Here the healing process was identical to bone elsewhere (Figure 2M,N,O).

#### Periosteum

The periosteum was frequently damaged during fracturing, and healing responses appeared to have exactly the same histological progression to those in the medulla. A periosteal reaction was sometimes seen in the absence of fracturing.

## Discussion

We present here a 32‐year study of histological findings and their relative time of occurrence in 169 fractures of known age from 52 infants. An initial tabulated data set based on 99 fractures from 26 infants was drawn up in 1999 and a rolling audit performed for a further 18 years.

This study was conducted to provide histopathologists examining fractures from cases of accidental or non‐accidental injury with a practical tool to age traumatic injuries of bones. While others have given valuable data on ageing fractures based on clinical observation,^9^, ^18^, ^19^, ^20^ this is the first study to provide background data tabulated by fracture age, histological feature and the proportion of cases showing that feature at defined time‐points following fracture.

The tabulated data can be considered an algorithm which, if one or more of the features described is recognised and then singly or in combination mapped onto the table, can be used to define an age range within which the fracture occurred. Similarly, if a feature is not present, the fracture has either not reached, or has passed, the age at which that specific feature becomes universally seen or disappears.

Data presented here indicate that all fractures appear to go through similar linear patterns of histological changes, with biological variation influencing differences in the rate of healing between individuals. Because it is not possible to know where within the biological spectrum an individual might lie fractures cannot be aged exactly, but our data indicate that a time range, during which the fracture occurred, can be given with a high degree of certainty. Age ranges for fractures of different ages can overlap; however, from examination of fractures of different known ages from the same individual, histology allows fractures to be clearly recognised as being of different ages.

In addition to producing a novel dating algorithm for infant fractures we report other new findings. We have shown:

- loss of osteocytes from bone at the periphery of very early fractures (1–2 h). All our observational data suggest that this cell death is energy‐requiring, conforming with current understanding of bone cell biology where osteocyte apoptosis is a necessary precursor of osteoclast‐driven bone remodelling;^26^, ^27^
- while metaphyseal fractures are difficult to age, if they involve bone the bone‐healing process matches that of fractures in other sites. Fractures involving only cartilage or non‐vascularised primary spongiosa cannot be aged with any certainty; and
- periosteum can be injured without fracturing and heals with the same processes over the same time‐frame as periosteal reactions associated with fractures.

## Conflict of interest

None. Professor Freemont has presented evidence in writing and in court based on these data.
